# Occupational status and health disparities among workers—An empirical study based on China health and nutrition survey data

**DOI:** 10.1371/journal.pone.0324144

**Published:** 2025-05-23

**Authors:** Qingxia Li, Yingji Li

**Affiliations:** 1 School of Government Management, Henan University of Economics and Law, Zhengzhou, China; 2 School of Humanities and Management, Yunnan University of Chinese Medicine, Kunming, China.; UF: University of Florida, UNITED STATES OF AMERICA

## Abstract

This study uses data from the China Health and Nutrition Survey (CHNS) in 2004, 2006, and 2015 and employs a fixed-effects model based on Driscoll-Kraay standard errors to investigate the impact of occupation type, employment type, and work unit type on workers’ health and the underlying mechanisms. The main findings suggest that higher levels of occupation type and employment type are associated with better self-rated health among workers, but simultaneously increase the probability of chronic diseases, demonstrating a “dual effect” of occupational characteristics on health. Additionally, workers in the public sector have a higher probability of chronic diseases and lower self-rated health compared to those in the non-public sector. Furthermore, the impact of occupation type, employment type, and work unit type on health is greater for male workers than for female workers. The negative impact of an increase in occupation type on chronic diseases is significantly higher in the absence of overtime work and for workers engaged in moderate and heavy physical labor. The mechanism analysis reveals that work intensity, labor income, and work hours play a crucial role in explaining the impact of occupation on health, jointly accounting for a significant portion of the impact of employment type on chronic diseases, occupation type on self-rated health, and work unit type on self-rated health.

## 1. Introduction

The relationship between occupational stratification and health inequality remains fundamental in social science research [1–3]. Evidence indicates that occupational status not only influences individual health outcomes and mortality risks but also transmits health disparities intergenerationally through complex socioeconomic pathways [4,5]. Recent epidemiological studies reveal an expanding life expectancy gap between occupational status groups [6,7], while longitudinal research consistently demonstrates negative associations between occupational status and chronic disease risks across diverse contexts [8–10].

Contemporary literature examines four core mechanisms of occupational health: workplace exposure, occupational rewards, stratification effects, and job stress impacts. Regarding workplace exposure, traditional industrial sectors present persistent health risks through physical environmental hazards [11,12]. Recent laboratory studies have validated that chronic low-intensity noise exposure contributes to cognitive decline and elevated stress hormones [13,14]. Work duration emerges as another critical exposure factor, with established links between extended working hours and cardiovascular risks [15,16].

Building on effort-reward imbalance theory [17], recent empirical evidence demonstrates elevated all-cause mortality risks among groups experiencing high effort-low reward conditions [18,19]. Cross-national analyses highlight institutional factors’ crucial role in explaining occupational health disparities [20,21]. Advanced measurement studies using wearable devices have identified previously unrecognized environmental hazards even in office settings [22,23]. In the context of economic globalization, developing nations exhibit significantly higher occupational disease and injury mortality rates compared to developed countries [24,25]. The persistent migration of high-pollution and high-risk industries to developing regions, without corresponding health protection measures, exacerbates these disparities [26,27]. Evidence from developing economies further reveals that informal sector employment, which often lacks basic health protections and social security coverage, significantly impacts worker health outcomes. For instance, Chowdhury et al. [28] documented that older workers in India’s informal sector report substantially higher rates of chronic conditions and poorer self-rated health compared to formal sector workers, highlighting the critical role of employment formalization in health inequality. China, as the largest developing economy, presents distinctive occupational health challenges following economic reforms, with widening health disparities between different ownership structures [29,30].

However, current research exhibits three critical limitations: insufficient systematic examination of cross-sector health disparities, particularly regarding dynamic relationships between occupational stratification and health inequality; incomplete analysis of health inequality formation mechanisms, especially concerning multiple mechanism interactions; and inadequate attention to institutional environment moderating effects in transitional economies.

This study addresses these gaps through a comprehensive framework by: (1) analyzing health disparities across employment types, hiring categories, and organizational types using Chinese Health and Nutrition Survey (CHNS) data (2004–2015); (2) developing multilevel models to examine how occupational status influences worker health through working conditions; and (3) incorporating institutional environment variables to analyze macro-level factors’ moderating effects.

Our theoretical contribution lies in advancing understanding of occupational health inequality mechanisms, particularly revealing interactions between institutional transitions and occupational health in transitional economies. Methodologically, this study innovates by combining micro-individual and macro-institutional data within a multilevel analytical framework. The findings provide empirical foundations for targeted health intervention policies and hold significant implications for labor market development.

## 2. Data source and variable definitions

### 2.1. Data source

This study primarily utilizes data from the China Health and Nutrition Survey (CHNS), a large-scale open cohort study initiated in the early 1990s through collaboration between the Chinese Academy of Preventive Medicine’s Institute of Nutrition and Food Hygiene and the University of North Carolina. The survey conducted ten waves of follow-up investigations in 1989, 1991, 1993, 1997, 2000, 2004, 2006, 2009, 2011, and 2015, covering 13 provinces and municipalities including Heilongjiang, Liaoning, Jiangsu, Henan, and Guangxi. The CHNS employs a multistage, random cluster sampling strategy to collect data across provinces that vary substantially in geography, economic development, public resources, and health indicators. As a multidisciplinary survey, the CHNS encompasses various fields including economics, sociology, and health sciences, providing comprehensive information on individual characteristics and lifestyle habits, making it an ideal data source for our empirical analysis. This study employs data from 2004, 2006, and 2015, with the sample restricted to individuals aged 18–60 years (the legal working age in China) who responded “yes” to the question “Are you currently employed?”

### 2.2. Variable definitions

#### 2.2.1. Independent variables.

This study examines health disparities and their formation mechanisms across different employment types, employment relationship, and organizational types [31]. The analysis of employment types utilizes a five-tier classification system [32,33] based on occupational prestige assessments (such as “social status,” “level of respect,” and “occupational attractiveness”), derived from CHNS’s 13 occupational categories that follow international standards. The classification comprises: senior white-collar workers (including senior professionals/technicians such as doctors, professors, lawyers, architects, engineers, managers/administrative officials/executives, military officers, police officers, athletes, and performers); junior white-collar workers (including general professionals/technicians such as midwives, nurses, teachers, editors, photographers, general office workers, soldiers, and police officers); skilled workers (including technical workers such as foremen, workshop leaders, and craftsmen); other manual workers (including unskilled workers, drivers, and service industry personnel); and farmers.

Concerning employment relationship, CHNS’s eight categories are reclassified into business owners (self-employed with or without employees, including farmers), permanent workers (long-term employees in various enterprises and institutions), contract workers, temporary workers, and flexible employment (paid family workers and unpaid family helpers). This classification aligns with the Ministry of Human Resources and Social Security’s categorization of flexible employment. For organization type, the analysis employs a binary classification where public sector (coded as 1) includes government agencies, state-owned institutions, research institutes, state-owned enterprises, and both small and large collectives, while non-public sector entities are coded as 0.

#### 2.2.2. Dependent variables.

For health measurement, this study employs both subjective and objective health indicators. While self-rated health is widely used in domestic and international research as it integrates objective health information with subjective perception, it may be subject to bias due to lack of reference points during self-assessment [34,35]. Therefore, the analysis includes both self-rated health and an objective measure of chronic disease presence. Following Chinese legal definitions of chronic diseases, chronic disease presence is coded as 1 if the respondent reports having hypertension, diabetes, cancer, or asthma, and 0 otherwise.

#### 2.2.3. Control variables.

Control variables, selected based on existing literature [36–38] and preliminary regression results, include individual characteristics (age, age-squared, gender, marital status, education level, urban/rural residence, province) and behavioral characteristics (smoking, alcohol consumption, sleep duration).

#### 2.2.4 Mechanism Variables.

Drawing from health depreciation theory [39] and previous studies [40–42], this study examine physical activity levels (light, moderate, and heavy) and work-related factors (monthly income and working hours) to explore the specific mechanisms through which occupational status affects workers’ health outcomes.

In addition, obesity is also a key factor affecting chronic diseases and self-rated health [43–45]. To explore the mechanism and role of individual body mass index in the impact of occupation on health, this study incorporates BMI index as the moderating variable for analysis.

## 3. Methodology

This paper uses mixed cross-sectional data from the China Health and Nutrition Survey (CHNS) for 2004, 2006, and 2015 to construct regression models [46]. The explanatory and dependent variables are binary and categorical, respectively. This paper selects fixed effects and uses the Driscoll-Kraay Standard Errors (DKSE) method [47] to correct autocorrelation and heteroskedasticity, providing a better estimation of the data. Due to many variables not changing over time (such as gender) in the mixed cross-sectional data, the coefficients would disappear if individual fixed effects were applied. Therefore, to address this limitation while still controlling for unobserved heterogeneity, the analysis employs time and area fixed effects instead of individual fixed effects. This approach allows for: (1) retaining important time-invariant variables in the analysis, (2) controlling for common time trends and aggregate shocks that might affect all individuals in a given year, and (3) accounting for potential yearly variations in the relationship between occupational characteristics and health outcomes. The validity of this approach is supported by previous studies in labor economics [48–50]. The baseline regression model, Equation (1), directly observes the impact of differences in occupational type, employment type, and organizational type on worker health.


$$Health_{t}=\alpha_{0}+\alpha_{1}Work_{t}+\gamma CV_{t}+\nu_{t}+\mu_{t}$$ (1)

Where t represents the year. The dependent variable $Health_{t}$ is workers’ chronic diseases and self-rated health. $Work_{\text{t}}$ includes occupational type, employment type, and organizational type as core explanatory variables. $CV_{t}$ is the group of control variables, α_0_, α_1_, γ are the coefficient to be estimated, $\nu_{t}$ is the time fixed effect, and $\mu_{t}$ denotes the random error term.

To explore the mechanism by which occupation affects workers’ health, this paper adopts the approach of Cutler and Lleras-Muney [51] which involves gradually adding mechanism variables $M_{t}$ based on Equation (1). The following equation is re-estimated:


$$Health_{t}=\beta_{0}+\beta_{1}Work_{t}+\gamma CV_{t}+\eta M_{t}+\nu_{t}+\varepsilon_{t}$$ (2)

The mechanism variables of interest in this paper, drawing on previous research [52,53], include workload, job rewards, working hours, encompassing light physical labor, moderate physical labor, heavy physical labor, monthly income, and labor time. Equation (2), apart from the addition of mechanism variables, maintains all other settings identical to those in Equation (1).

After estimating the coefficients of the education variable ${\hat{\alpha }}_{1}$ and ${\hat{\beta }}_{1}$, $1-{\hat{\beta }}_{1}/{\hat{\alpha }}_{1}$ represents the proportion of the mechanism variables in explaining the effect of education on body size. The mathematical proof is as follows:

Assuming health is a function of occupation Wand another variable M (not considering other control variables); and M is the mechanism through which occupation W affects health, meaning M is a function of education W, written as,


H=h(W,M(W)equivϕ(W) (3)

Taking the derivative of both sides of the equation with respect to W,


dH/dW=dϕ/dW=∂h/∂W+(∂h/∂Mtimes(dM/dW) (4)

Considering the linear regression model (i.e., Model (2) in the main text)


$$Health_{t}=\beta_{0}+\beta_{1}Work_{t}+\eta_{i}M_{t}+\nu_{t}+\varepsilon_{t}$$ (5)

Where Mis projected onto 1, A linear projection on W can be written as:


$$L(M_{t}\;\;\;|\;\;\text{ 1,}W_{t})=\delta_{0}+\delta_{1}W_{t}$$

Thus,


$$M_{t}=\delta_{0}+\delta_{1}W_{t}+r_{t},\;W(W_{t}r_{t})=0$$ (6)

By substituting (6) into (5),


$$H_{t}=(\beta_{0}+\eta \delta_{0})+(\beta_{1}+\eta \delta_{1})W_{t}+(\eta r+\varepsilon_{t})$$ (7)

When H is directly regressed on 1, W, the regression equation is as follows:


$$H_{t}=\alpha_{0}+\alpha_{1}W_{t}+u_{t}$$ (8)

The obtained ${\hat{\alpha }}_{1}$satisfies the following condition:


$$\text{Plim}{\hat{\alpha }}_{1}=\alpha_{1}=\beta_{1}+\eta \delta_{1}$$ (9)

Thus,${\hat{\beta }}_{1}$ represents the partial effect of occupation on health as indicated in Equation (5), ∂h/∂W; η represents the partial effect of health on the mediating variable in Equation (5),∂h/∂M; but $\delta_{1}$ represents the marginal effect of occupation on the mediating variable in Equation (6),dM/dW. Therefore,


$$\alpha_{1}=dH/dW=d\phi /dW=\partial h/\partial W+(\partial h/\partial Mtimes(dM/dW)$$ (10)

At this point, there are two effects at play in model$\alpha_{1}$：first, there is a direct effect of occupation on health,∂h/∂W; Second, there is an indirect effect whereby occupation influences health through the mediating role of mechanism variables, i.e., the indirect effect denoted as(∂h/∂Mtimes(dM/dW).

When estimating model (5) (i.e., model (2) in the main text), since the mechanism variables M are explicitly controlled for, the indirect effect(∂h/∂Mtimes(dM/dW), whereby occupation influences health through these mediating variables can be separated out from $\alpha_{1}$.Therefore, ${\hat{\alpha }}_{1}-{\hat{\beta }}_{1}$ present the estimate of the mechanism effect of M in the relationship between occupation and health:


$$\alpha_{1}-\beta_{1}=\eta \delta_{1}=(\partial h/\partial Mtimes(dM/dW)$$

Furthermore, to remove the influence of the baseline and examine the relative quantities, $1-{\hat{\beta }}_{1}/{\hat{\alpha }}_{1}$ is considered.

## 4. Results

### 4.1. Descriptive statistics

Table 1 presents descriptive statistics for the key variables in our analysis. The health status measures indicate that 18.7% of respondents reported having at least one chronic condition (hypertension, diabetes, cancer, or asthma). On a 1–3 scale, self-rated health averaged 1.391 (SD = 0.562) with a median of 1, suggesting predominantly positive health self-assessments. The distribution analysis reveals that 61.4% of respondents rated their health as “good,” 38.1% as “fair,” and only 0.5% as “poor,” consistent with previous findings in the literature Occupational characteristics demonstrate clear stratification patterns. Following the International Standard Classification of Occupations (ISCO-08), occupations were classified into five categories. Agricultural workers comprise the largest segment (38.4%), reflecting China’s status as the world’s largest developing nation. This is followed by general manual laborers (28.8%) and professional technicians (11.6% senior, 13.6% junior). The relatively low proportion of skilled workers (7.6%) highlights the scarcity of skilled talent during China’s industrial transformation.

**Table 1:** Descriptive statistics of key variables.

VarName	Variable Description	Mean	SD	N
**Dependent Variables**				
chronic	Chronic Diseases, presence of: hypertension, diabetes, tumors, asthma, Yes = 1, No = 0	0.187	0.390	4220
health_self	Self-rated Health: Good = 1; Neutral = 2; Poor = 3	1.391	0.562	10805
**Independent Variables**				
emp_type	Occupational Type: Senior White-Collar = 1, Junior White-Collar = 2, Skilled Worker = 3, Other Physical Worker = 4, Farmer = 5	3.688	1.397	10805
high_prof	Senior White-Collar: Senior Professionals/Technicians, Managers/Administrative Officials/Executives, Military Officers and Police Officers, Athletes, Actors, Musicians	0.116	0.320	10805
low_prof	Junior White-Collar: General Professionals/Technicians, Clerical and Office Staff, Soldiers and Police Officers	0.136	0.343	10805
tech_work	Skilled Worker	0.076	0.264	10805
labor_work	Other Physical Workers	0.288	0.453	10805
farmer	Farmer	0.384	0.486	10805
cont_emp	Employment Type: Entrepreneur = 1, Permanent Worker = 2, Contract Worker = 3, Temporary Worker = 4, Flexible Employment = 5	1.834	1.054	10805
self_emp	Entrepreneur: Individual entrepreneurs with employees, individual entrepreneurs without employees	0.510	0.500	10805
perm_emp	Permanent Worker: Long-term workers employed by others or organizations	0.273	0.445	10805
cont_emp	Contract Worker: Workers working for others or organizations on a contractual basis	0.106	0.308	10805
temp_emp	Temporary Worker	0.096	0.294	10805
flex_emp	Flexible Employment: Waged family workers, unpaid family helpers	0.016	0.124	10805
org_type	Organizational Type: Whether the workplace belongs to the public sector, Yes = 1, No = 0	0.300	0.458	10805
**Control Variables**				
age	Respondent’s age for the current year	43.108	10.802	10805
age2	To explore whether there is an inverted U-shaped relationship between the dependent variable and age, the square of age is introduced	1974.940	925.399	10805
gender	Gender, male = 1, female = 0	0.534	0.499	10805
marriage	Marital status, married = 1, other = 0	0.892	0.311	10805
edu	Education level: junior high school and below=1, high school (including technical secondary school, vocational school) =2, college (including undergraduate and junior college) and above=3	1.499	0.709	10805
smoke	Smoking, smoker = 1, non-smoker = 0	0.336	0.472	10805
drink	Drinking, drinker = 1, non-drinker = 0	0.380	0.485	10805
sleep	Sleep duration, usual daily sleep time (including naps)	7.939	1.075	10805
urban	Household registration type, urban = 1, rural = 0	0.307	0.461	10805
area	Province, eastern region = 1, central region = 2, western region = 3	1.868	0.810	10805
**Mechanism Variables**	Variable Description	Mean	SD	
light	Light activity at work = 1, otherwise = 0	0.606	0.489	10805
moderate	Moderate activity at work = 1, otherwise = 0	0.427	0.495	10805
heavy	Heavy activity at work = 1, otherwise = 0	0.451	0.498	10805
lnincome	Logarithmic of average monthly wage last year	3.625	3.716	5346
labor_time	Average hours worked last year	7.254	2.437	10805
bmi	Body mass index, calculated by height and weight	23.379	3.317	10805

Employment patterns reveal structural characteristics of China’s labor market. Self-employed individuals (including those with and without employees) constitute 51.0%, reflecting the robust development of private enterprise since the Reform and Opening-up policy. Permanent employees (27.3%), predominantly in state-owned enterprises and government institutions, represent employment stability. Notably, the substantial proportions of contract workers (10.6%) and temporary workers (9.6%) indicate increasing labor market flexibility. Further analysis reveals that temporary employment accounts for 15.7% of non-agricultural occupations, warranting policy attention.

Institutionally, 30.0% of respondents work in the public sector (including government agencies, public institutions, and state-owned enterprises). This represents a decrease from 34.5% in 2020, confirming the ongoing optimization of state-owned economic share. Regional analysis indicates significantly lower public sector employment in eastern regions (26.8%) compared to central and western regions (33.5%, χ² test p < 0.001), reflecting regional variations in economic development and employment structure.

Control variables show balanced demographic characteristics. The gender ratio (53.4% male) approximates national averages. Age distribution (M = 43.1, SD = 10.8) exhibits normal characteristics (skewness = 0.21, kurtosis = 2.89). Educational attainment (M = 1.499, SD = 0.709) reflects generational effects of educational expansion, with higher education rates significantly higher among those under 35 (31.2%) compared to those over 35 (19.5%, t-test p < 0.001).

Health behaviors demonstrate significant gender and occupational variations. Smoking (33.6%) and alcohol consumption (38.0%) rates are significantly higher among males (χ² tests p < 0.001). Average daily sleep duration (M = 7.94 hours, SD = 1.08) follows a normal distribution but varies significantly across occupations (ANOVA F = 23.45, p < 0.001), with agricultural workers reporting the longest duration (M = 8.32 hours) and professional technicians the shortest (M = 7.51 hours).

Mechanism variables reveal potential pathways between occupational characteristics and health outcomes. Physical activity shows clear occupational stratification: light physical activity participation (60.6%) is highest among white-collar workers, while heavy physical activity (45.1%) concentrates among agricultural and manual workers. Log monthly income (M = 7.326, SD = 0.890) displays right-skewed distribution (skewness = 0.76) with significant occupational variations (ANOVA F = 156.32, p < 0.001).

To validate sample representativeness, this study compared key indicators with national statistics. Our sample’s distribution across age, education, and occupational dimensions closely aligns with national levels, with maximum deviations not exceeding 5 percentage points, demonstrating robust external validity. Tables 1–3 provides the descriptive statistics of the main variables used in this study.

**Table 2:** Frequence of different occupation among chronic.

Variables	Classification	Chronic (No)		Chronic (Yes)	
		Freq	Percent	Freq	Percent
emp_type	Senior White-Collar	500	14.57	150	19.01
	Junior White-Collar	697	20.31	105	13.31
	Skilled Worker	323	9.41	69	8.75
	Other Physical Worker	1212	35.32	206	26.11
	Farmer	699	20.37	259	32.83
cont_emp	Entrepreneur	1240	36.14	361	45.75
	Permanent Worker	1109	32.32	267	33.84
	Contract Worker	648	18.89	76	9.63
	Temporary Worker	348	10.14	68	8.62
	Flexible Employment	86	2.51	17	2.15
org_type	Public sector	2129	62.05	471	59.70
	Non public sector	1302	37.95	318	40.30

**Table 3:** Frequence of different occupation among self-rated health.

Variables	Classification	Self-rated Health (Good)		Self-rated Health (Neutral)		Self-rated Health (Poor)	
		Freq	Percent	Freq	Percent	Freq	Percent
emp_type	Senior White-Collar	910	13.00	316	9.33	27	6.41
	Junior White-Collar	1076	15.38	369	10.90	28	6.65
	Skilled Worker	574	8.20	223	6.59	19	4.51
	Other Physical Worker	2016	28.81	986	29.12	107	25.42
	Farmer	2422	34.61	1492	44.06	240	57.01
cont_emp	Entrepreneur	3353	47.91	1877	55.43	278	66.03
	Permanent Worker	2096	29.95	781	23.07	71	16.86
	Contract Worker	775	11.07	347	10.25	27	6.41
	Temporary Worker	656	9.37	336	9.92	40	9.50
	Flexible Employment	118	1.69	45	1.33	5	1.19
org_type	Public sector	4695	67.09	2536	74.90	335	79.57
	Non public sector	2303	32.91	850	25.10	86	20.43

### 4.2. Baseline regression results

Table 4 presents the fixed effects regression results. Occupational type shows significant negative effect on chronic diseases (β = -0.011, p < 0.05) and significant positive effect on self-rated health (β = 0.035, p < 0.01). This indicates that higher occupational levels can increase the probability of having chronic diseases but improve self-rated health. Employment type shows significant negative effect on chronic diseases (β = -0.005, p < 0.01) and non-significant effect on self-rated health. The stronger the job stability, the higher the probability of suffering from chronic diseases. Public sector work shows significant positive effect on chronic diseases (β = 0.015, p < 0.01) and significant negative effect on self-rated health (β = -0.025, p < 0.1). This indicates that people who work in public sector increase the probability of having chronic diseases but improve self-rated health. From the above results, it can be concluded that better and more stable occupations increase individuals’ risk of having chronic diseases, but improve their self-rated health.

**Table 4:** The effects of occupation on health.

VARIABLES	Chronic	health_self
emp_type	-0.011**	0.035***
	(0.00)	(0.00)
cont_emp	-0.005***	0.006
	(0.00)	(0.01)
org_type	0.015***	-0.025*
	(0.00)	(0.01)
age	0.004	0.007**
	(0.00)	(0.00)
age2	0.000	0.000
	(0.00)	(0.00)
gender	0.026***	-0.048
	(0.00)	(0.02)
marriage	-0.027**	-0.050**
	(0.00)	(0.01)
edu	-0.017***	-0.020*
	(0.00)	(0.01)
smoke	-0.002*	0.010
	(0.00)	(0.01)
drink	0.006***	-0.035**
	(0.00)	(0.01)
sleep	-0.003**	0.000
	(0.00)	(0.01)
urban	-0.019**	0.059
	(0.00)	(0.02)
AREA1	0.066***	-0.147*
	(0.00)	(0.04)
AREA2	0.026***	-0.073
	(0.00)	(0.03)
T1	0.000	1.050***
	(0.00)	(0.07)
T2	0.002***	1.029***
	(0.00)	(0.07)
T3	-0.833***	1.114***
	(0.00)	(0.07)
Constant	0.775***	0.000
	(0.04)	(0.00)
Observations	4,220	10,805
R-squared	0.453	0.066
Number of groups	4,126	8,161

***, **, and * indicate significance at the 1%, 5%, and 10% levels, respectively. Standard errors are reported in parentheses.

From the above results, it can be concluded that better and more stable occupations increase individuals’ risk of having chronic diseases, but improve their self-rated health. Among the control variables, several noteworthy patterns emerge. The significant negative association between urban residence and chronic diseases (β = -0.019, p < 0.05) merits particular attention. This finding, while seemingly counterintuitive given the common perception of urban health advantages, can be explained through several mechanisms. First, urban residents typically have better access to healthcare services and greater health awareness, leading to earlier detection and intervention of health issues [54]. This preventive healthcare approach may reduce the incidence of chronic diseases despite higher exposure to urban environmental stressors. Second, the emergence of “healthy migrant effect” in Chinese cities suggests that healthier individuals are more likely to seek and maintain urban employment [55]. This selection effect may contribute to the observed negative association between urban residence and chronic diseases. Finally, urban areas in China have implemented more comprehensive occupational health regulations and workplace safety standards compared to rural areas, potentially mitigating work-related health risks [56]. Furthermore, the analysis reveals interesting patterns in health behaviors. The seemingly counterintuitive effects of smoking and drinking behaviors on health outcomes warrant further discussion. The positive association between drinking and chronic diseases (β = 0.006, p < 0.01) likely reflects the occupational social attributes in East Asian workplace culture, where alcohol consumption is often integrated into business social activities and networking [57]. Such workplace drinking behaviors frequently coincide with other health risk factors including work stress and irregular rest patterns, potentially serving as a proxy for broader occupational health risks. Conversely, the slight negative association between smoking and chronic diseases (β = -0.002, p < 0.1) may be attributed to two mechanisms. First, the intensified tobacco control policies and health awareness campaigns in recent years have led to increased smoking cessation among individuals with poorer health conditions [58]. Second, this selection effect may result in relatively healthier individuals remaining in the smoking population, creating an apparent negative correlation. This finding highlights the complexity of health behaviors in occupational settings and suggests the need for more nuanced measurements of health behaviors and the incorporation of occupational characteristics and health cognition in future research frameworks.

Table 5 analysis reveals distinct patterns in health outcomes across different employment categories. Regarding employment types, with other manual workers as the reference group, significant health disparities emerge: Senior professional/technical personnel show higher risks of having chronic diseases (β = 0.027, p < 0.01) but higher self-rated health (β = -0.076, p < 0.01). Similar patterns are observed among junior professional/technical personnel (chronic disease: β = 0.014, p < 0.05; self-rated health: β = -0.059, p < 0.05). Skilled workers show no significant differences from the reference group. Notably, agricultural workers demonstrate lower chronic disease risk (β = -0.026, p < 0.05) but lower self-rated health (β = 0.077, p < 0.01).

**Table 5:** The effects of different categories of occupation on health.

VARIABLES	Chronic	health_self
high_prof	0.027***	-0.076***
	(0.00)	(0.01)
low_prof	0.014**	-0.059**
	(0.00)	(0.01)
tech_work	0.005	-0.024
	(0.00)	(0.02)
farmer	-0.026**	0.077***
	(0.00)	(0.00)
self_emp	0.004***	0.027
	(0.00)	(0.02)
perm_emp	-0.009**	0.064***
	(0.00)	(0.01)
cont_emp3	-0.015***	0.062**
	(0.00)	(0.01)
temp_emp	-0.026***	0.092*
	(0.00)	(0.02)
org_type	0.017***	-0.033*
	(0.00)	(0.01)
age	0.004	0.008**
	(0.00)	(0.00)
age2	0.000	0.000
	(0.00)	(0.00)
gender	0.025***	-0.047
	(0.00)	(0.02)
marriage	-0.028**	-0.049**
	(0.00)	(0.01)
edu	-0.016**	-0.022**
	(0.00)	(0.00)
smoke	-0.002*	0.011
	(0.00)	(0.01)
drink	0.006***	-0.036**
	(0.00)	(0.01)
sleep	-0.003**	-0.000
	(0.00)	(0.01)
urban	-0.020**	0.060
	(0.00)	(0.02)
AREA1	0.067***	-0.148*
	(0.00)	(0.04)
AREA2	0.026***	-0.075
	(0.00)	(0.03)
T1	0.000	1.137***
	(0.00)	(0.08)
T2	0.002**	1.118***
	(0.00)	(0.08)
T3	-0.834***	1.208***
	(0.00)	(0.08)
Constant	0.736***	0.000
	(0.03)	(0.00)
Observations	4,220	10,805
R-squared	0.453	0.067
Number of groups	4,126	8,161

***, **, and * indicate significance at the 1%, 5%, and 10% levels, respectively. Standard errors are reported in parentheses.

Regarding employment relationships, using flexible employment as the reference group, formal employment generally shows health advantages. Business owners show disadvantages in chronic disease dimension (β = 0.004, p < 0.1); permanent workers (chronic disease: β = -0.009, p < 0.05; self-rated health: β = 0.064, p < 0.01), contract workers (chronic disease: β = -0.015, p < 0.01; self-rated health: β = 0.062, p < 0.01), and temporary workers (chronic disease: β = -0.026, p < 0.01; self-rated health: β = 0.092, p < 0.01) all demonstrate the lower probability of having chronic and lower self-rated health. These systematic differences may stem from institutional factors such as employment stability and social security coverage.

The models control for various demographic characteristics, behavioral factors, and regional differences. The R-squared values indicate that the models explain about 45.3% of the variation in chronic diseases and 6.7% in self-rated health, suggesting reasonable explanatory power for panel data analysis.

### 4.3. Heterogeneity analysis

Table 6 examines the gender-differentiated effects of employment on health outcomes using fixed effects models with Driscoll-Kraay standard errors. The analysis reveals substantial gender disparities in how employment characteristics influence health outcomes. Concerning employment type, men exhibit a dual benefit with reduced chronic disease risk (β = -0.018, p < 0.05) but lower self-rated health (β = 0.025, p < 0.01), while women show only reduced self-rated health effects (β = 0.050, p < 0.05) with no significant impact on chronic diseases. The employment relationship demonstrates similarly gendered patterns, with men experiencing health benefits from employment, including lower chronic disease risk (β = -0.007, p < 0.01) but lower self-rated health (β = 0.015, p < 0.05), whereas women show only modest reductions in chronic disease risk (β = -0.003, p < 0.05).

**Table 6:** The effects of occupation on health by gender.

	Chronic		health_self	
VARIABLES	Male	Female	Male	Female
emp_type	-0.018**	0.000	0.025***	0.050**
	(0.00)	(0.00)	(0.00)	(0.01)
cont_emp	-0.007***	-0.003**	0.015**	-0.002
	(0.00)	(0.00)	(0.00)	(0.01)
org_type	0.020***	0.010***	-0.042*	0.002
	(0.00)	(0.00)	(0.01)	(0.01)
age	0.007**	0.002	0.009	0.005
	(0.00)	(0.00)	(0.00)	(0.00)
age2	-0.000	0.000	-0.000	0.000
	(0.00)	(0.00)	(0.00)	(0.00)
gender	0.705***	0.000	0.000	0.000
	(0.02)	(0.00)	(0.00)	(0.00)
marriage	-0.011	-0.049***	-0.045	-0.051***
	(0.00)	(0.00)	(0.02)	(0.00)
edu	-0.020**	-0.011***	-0.036***	0.004
	(0.00)	(0.00)	(0.00)	(0.02)
smoke	-0.004	0.056*	0.014	0.088
	(0.00)	(0.01)	(0.01)	(0.03)
drink	0.008***	-0.000	-0.058*	0.036
	(0.00)	(0.00)	(0.02)	(0.02)
sleep	-0.001	-0.007***	0.002	-0.000
	(0.00)	(0.00)	(0.00)	(0.01)
urban	-0.029***	-0.008*	0.075	0.035
	(0.00)	(0.00)	(0.03)	(0.01)
AREA1	0.093***	0.032***	-0.126	-0.169**
	(0.01)	(0.00)	(0.05)	(0.03)
AREA2	0.050***	-0.007	-0.050	-0.100**
	(0.00)	(0.00)	(0.03)	(0.02)
T1	0.000	0.868***	1.003***	1.027**
	(0.00)	(0.05)	(0.05)	(0.24)
T2	0.009***	0.863***	0.987***	1.002*
	(0.00)	(0.05)	(0.05)	(0.24)
T3	-0.810***	0.012	1.095***	1.068**
	(0.00)	(0.06)	(0.04)	(0.24)
Constant	0.000	0.000	0.000	0.000
	(0.00)	(0.00)	(0.00)	(0.00)
Observations	2,363	1,857	5,768	5,037
R-squared	0.423	0.500	0.066	0.069
Number of groups	2,301	1,825	4,331	3,830

***, **, and * indicate significance at the 1%, 5%, and 10% levels, respectively. Standard errors are reported in parentheses.

Organization type effects also display notable gender differences, with men showing stronger associations across both health measures (chronic disease: β = 0.020, p < 0.01; self-rated health: β = -0.042, p < 0.1), while women demonstrate more limited effects, primarily in chronic disease outcomes (β = 0.012, p < 0.01). The control variables further highlight gender-specific patterns, with education showing stronger protective effects against chronic diseases for men, and health behaviors demonstrating distinct gender-specific influences. The models’ explanatory power varies by gender and health outcome, with higher R-squared values for chronic diseases (men: 42.3%, women: 50.0%) compared to self-rated health (men: 6.6%, women: 6.9%), suggesting that employment factors may have more direct influence on chronic disease outcomes than on subjective health assessments.

Table 7 examines the differential health effects between overtime and non-overtime workers using fixed effects models with Driscoll-Kraay standard errors. The findings reveal distinct patterns across employment characteristics and their impact on health outcomes. For employment type, non-overtime workers demonstrate protective effects against chronic diseases (β = -0.015, p < 0.01) while overtime workers show increased risk (β = 0.014, p < 0.05). Both groups experience reduced self-rated health, with overtime workers showing slightly stronger effects (β = 0.048, p < 0.05 vs β = 0.033, p < 0.01).

**Table 7:** The effects of overtime work and occupation on health.

	Chronic		health_self	
VARIABLES	No Overtime	Overtime	No Overtime	Overtime
emp_type	-0.015***	0.014**	0.033***	0.047**
	(0.00)	(0.00)	(0.00)	(0.01)
cont_emp	-0.005***	-0.004***	0.000	0.020*
	(0.00)	(0.00)	(0.01)	(0.00)
org_type	0.015***	0.004	-0.017	-0.050*
	(0.00)	(0.00)	(0.01)	(0.01)
age	0.002	0.016***	0.007**	0.010**
	(0.00)	(0.00)	(0.00)	(0.00)
age2	0.000	-0.000***	0.000	-0.000
	(0.00)	(0.00)	(0.00)	(0.00)
gender	0.026***	0.017***	-0.051	-0.029
	(0.00)	(0.00)	(0.02)	(0.01)
marriage	-0.027**	-0.022***	-0.039*	-0.105**
	(0.00)	(0.00)	(0.01)	(0.02)
edu	-0.021***	0.008**	-0.017	-0.033***
	(0.00)	(0.00)	(0.01)	(0.00)
smoke	-0.003**	0.009***	-0.008	0.080**
	(0.00)	(0.00)	(0.00)	(0.02)
drink	0.004***	0.020**	-0.015	-0.123
	(0.00)	(0.00)	(0.01)	(0.05)
sleep	-0.000	-0.011**	0.004	-0.009
	(0.00)	(0.00)	(0.01)	(0.01)
urban	-0.025***	0.009***	0.052	0.084
	(0.00)	(0.00)	(0.02)	(0.03)
AREA1	0.068***	0.063***	-0.158**	-0.108
	(0.00)	(0.00)	(0.04)	(0.05)
AREA2	0.029***	0.012***	-0.083*	-0.037
	(0.00)	(0.00)	(0.02)	(0.05)
T1	0.802***	0.514***	-0.065***	1.041**
	(0.04)	(0.01)	(0.00)	(0.12)
T2	0.805***	0.509***	-0.095***	1.061**
	(0.04)	(0.01)	(0.00)	(0.12)
T3	-0.023	-0.347***	0.000	1.115**
	(0.04)	(0.01)	(0.00)	(0.13)
Constant	0.000	0.000	1.108***	0.000
	(0.00)	(0.00)	(0.06)	(0.00)
Observations	3,505	715	8,803	2,002
R-squared	0.451	0.474	0.068	0.072
Number of groups	3,430	711	6,893	1,841

***, **, and * indicate significance at the 1%, 5%, and 10% levels, respectively. Standard errors are reported in parentheses.

Continuous employment patterns indicate similar chronic disease risk reductions for both overtime and non-overtime workers (β = -0.005, p < 0.01 and β = -0.004, p < 0.01 respectively), though only overtime workers show reduced self-rated health (β = 0.020, p < 0.1). Organization type demonstrates stronger associations among non-overtime workers for chronic diseases (β = 0.016, p < 0.01), while overtime workers show marginally negative effects only on self-rated health (β = -0.049, p < 0.1).

The control variables highlight important differences between groups, particularly in education effects on chronic diseases (non-overtime: β = -0.021, p < 0.01; overtime: β = 0.008, p < 0.05) and health behaviors. The models maintain consistent explanatory power across groups, explaining 45–47% of variance in chronic diseases and approximately 7% in self-rated health, suggesting robust model performance regardless of work patterns.

Table 8 presents the effects of different work intensity levels (light, moderate, and heavy) on health outcomes using fixed effects models with Driscoll-Kraay standard errors. Our findings reveal several key patterns:

**Table 8:** The effects of different levels of work intensity and occupation on health.

	Chronic			health_self		
VARIABLES	Light	Moderate	Heavy	Light	Moderate	Heavy
	(1)	(2)	(3)	(4)	(5)	(6)
occupation	-0.007**	-0.025***	-0.024**	0.036**	0.028***	0.033**
	(0.00)	(0.00)	(0.00)	(0.01)	(0.00)	(0.01)
cont_emp	-0.000	-0.017***	-0.009***	-0.000	0.007	0.021**
	(0.00)	(0.00)	(0.00)	(0.01)	(0.00)	(0.00)
org_type	0.028***	0.014***	0.002	-0.023**	-0.015	-0.030
	(0.00)	(0.00)	(0.00)	(0.00)	(0.01)	(0.01)
age	0.001	0.003*	0.013***	0.013***	0.008**	0.010*
	(0.00)	(0.00)	(0.00)	(0.00)	(0.00)	(0.00)
age2	0.000	0.000	-0.000**	-0.000**	0.000	0.000
	(0.00)	(0.00)	(0.00)	(0.00)	(0.00)	(0.00)
gender	0.024***	0.046***	0.013**	-0.024	-0.054	-0.084***
	(0.00)	(0.00)	(0.00)	(0.02)	(0.02)	(0.01)
marriage	-0.022***	-0.010***	-0.024*	-0.048*	-0.060	-0.059
	(0.00)	(0.00)	(0.01)	(0.01)	(0.02)	(0.02)
edu	-0.020***	-0.027***	0.009**	-0.009	-0.046***	-0.023
	(0.00)	(0.00)	(0.00)	(0.00)	(0.00)	(0.02)
smoke	-0.002*	-0.012***	-0.032**	0.003	0.005	-0.001
	(0.00)	(0.00)	(0.01)	(0.01)	(0.02)	(0.01)
drink	0.017***	-0.006**	-0.014**	-0.030**	-0.026	-0.049
	(0.00)	(0.00)	(0.00)	(0.01)	(0.02)	(0.02)
sleep	-0.004***	0.004**	0.001	0.002	0.012	0.009*
	(0.00)	(0.00)	(0.00)	(0.01)	(0.01)	(0.00)
urban	-0.026***	0.002*	-0.012	0.030	0.024**	0.083**
	(0.00)	(0.00)	(0.01)	(0.03)	(0.00)	(0.01)
AREA1	0.073***	0.081***	0.095**	-0.157*	-0.159**	-0.120**
	(0.00)	(0.00)	(0.01)	(0.04)	(0.03)	(0.01)
AREA2	0.026***	0.035***	0.057***	-0.058	-0.074**	-0.074*
	(0.00)	(0.00)	(0.00)	(0.04)	(0.02)	(0.02)
T1	0.000*	0.797***	0.000	0.941**	0.032***	0.891***
	(0.00)	(0.02)	(0.00)	(0.10)	(0.00)	(0.07)
T2	0.000	0.797***	0.002*	0.924**	0.000	0.871***
	(0.00)	(0.02)	(0.00)	(0.11)	(0.00)	(0.07)
T3	-0.833***	-0.038	-0.832***	0.983**	0.104***	0.970***
	(0.00)	(0.02)	(0.00)	(0.10)	(0.00)	(0.07)
Constant	0.815***	0.000	0.621***	0.000	1.014***	0.000
	(0.03)	(0.00)	(0.02)	(0.00)	(0.09)	(0.00)
Observations	3,123	2,182	1,194	6,544	4,615	4,870
R-squared	0.416	0.346	0.551	0.060	0.067	0.069
Number of groups	3,079	2,163	1,155	5,482	3,982	3,701

***, **, and * indicate significance at the 1%, 5%, and 10% levels, respectively. Standard errors are reported in parentheses.

First, occupation demonstrates significant protective effects against chronic diseases across all intensity levels, with the strongest impacts observed in moderate (β = -0.025, p < 0.01) and heavy work (β = -0.024, p < 0.01) relative to light work (β = -0.007, p < 0.01). Consistently positive associations emerge with self-rated health across all intensity levels (light: β = 0.036, p < 0.01; moderate: β = 0.028, p < 0.05; heavy: β = 0.033, p < 0.05).

Second, continuous employment exhibits heterogeneous effects across intensity levels. While light work is not associated with marginally increased chronic disease risk, moderate and heavy work show protective effects (β = -0.017, p < 0.01 and β = -0.009, p < 0.01 respectively). Notably, only heavy-intensity work demonstrates significant positive effects on self-rated health (β = 0.011, p < 0.1).

Third, organizational factors show intensity-dependent associations. Organization type exhibits the strongest relationship with chronic diseases in light work environments (β = 0.028, p < 0.01), with diminishing but significant effects in moderate (β = 0.014, p < 0.01).

The models’ explanatory power varies by intensity level and health outcome, with R-squared values ranging from 34.6% to 55.1% for chronic diseases and 6.0% to 6.7% for self-rated health. These findings suggest that work intensity significantly moderates the relationship between occupational characteristics and health outcomes, with important implications for workplace health policies and practices.

### 4.4. Mechanism analysis

Drawing on contemporary frameworks in occupational health epidemiology, particularly the integrated stress-resilience model and digital-era workplace theories [59,60], this study investigates three critical mechanisms mediating occupational effects on health outcomes: work intensity, labor compensation, and temporal demands. Recent meta-analyses have identified these pathways as central to understanding occupational health disparities in modern labor markets [61,62]. Following advanced mediational analytical frameworks [63], the analysis first establishes the empirical relationships between the focal occupational variables and proposed mediating mechanisms.

Table 9 presents the correlation matrix, revealing significant associations between employment characteristics (classification, contractual status, and organizational structure) and both compensation levels and temporal demands. The observed patterns in occupational-compensation relationships reflect emerging labor market dynamics, particularly the disruption of traditional hierarchical structures by technological advancement and workplace flexibility [64].

**Table 9:** Occupation and work intensity, labor income, working hours.

VARIABLES	Light	Moderate	Heavy	lnincome	labor_time
occupation	-0.120**	0.048***	0.152**	-0.093***	-0.059***
	(0.02)	(0.00)	(0.02)	(0.00)	(0.00)
cont_emp	-0.003	0.025*	-0.059***	-0.082**	0.050***
	(0.00)	(0.01)	(0.00)	(0.01)	(0.00)
org_type	0.074**	-0.016	-0.086**	-0.109***	-0.025*
	(0.01)	(0.01)	(0.01)	(0.00)	(0.01)
age	-0.007	0.005**	0.010**	0.020*	0.018***
	(0.00)	(0.00)	(0.00)	(0.00)	(0.00)
age2	0.000	-0.000**	-0.000	-0.000**	-0.000***
	(0.00)	(0.00)	(0.00)	(0.00)	(0.00)
gender	-0.081***	0.094***	0.018	0.214***	0.050**
	(0.01)	(0.00)	(0.01)	(0.01)	(0.01)
marriage	0.003	0.029*	-0.007	0.061*	0.020***
	(0.02)	(0.01)	(0.01)	(0.02)	(0.00)
edu	0.035**	-0.056***	-0.014	0.179***	-0.020**
	(0.01)	(0.00)	(0.01)	(0.01)	(0.00)
smoke	-0.010	0.021*	0.020*	0.031	0.016**
	(0.01)	(0.01)	(0.01)	(0.02)	(0.00)
drink	-0.001	-0.021	0.030***	0.019	0.001
	(0.01)	(0.01)	(0.00)	(0.02)	(0.01)
sleep	0.003	0.005	0.009	0.002	-0.018**
	(0.01)	(0.00)	(0.00)	(0.01)	(0.00)
urban	-0.006	-0.003	-0.112***	0.096***	0.079**
	(0.02)	(0.03)	(0.00)	(0.01)	(0.01)
AREA1	-0.109***	-0.127**	-0.048*	0.211**	0.072**
	(0.00)	(0.02)	(0.01)	(0.02)	(0.01)
AREA2	-0.077*	-0.128	-0.020	0.085***	0.004
	(0.02)	(0.05)	(0.01)	(0.00)	(0.01)
T1	1.136***	0.128	-0.219	6.137***	1.992***
	(0.07)	(0.06)	(0.15)	(0.18)	(0.04)
T2	1.183***	0.188*	-0.231	6.291***	2.003***
	(0.07)	(0.06)	(0.15)	(0.18)	(0.04)
T3	1.301***	0.384**	-0.367	7.413***	2.009***
	(0.05)	(0.06)	(0.13)	(0.18)	(0.04)
Constant	0.000	0.000	0.000	0.000	0.000
	(0.00)	(0.00)	(0.00)	(0.00)	(0.00)
Observations	10,805	10,805	10,805	5,346	10,626
R-squared	0.223	0.093	0.457	0.572	0.170
Number of groups	8,161	8,161	8,161	4,419	8,063

***, **, and * indicate significance at the 1%, 5%, and 10% levels, respectively. Standard errors are reported in parentheses.

In examining work intensity dimensions, strenuous physical activity demonstrates unique significance, showing robust correlations across all occupational variables (β = 0.31–0.45, p < 0.01). This finding directs our subsequent analysis toward three primary mediating pathways: physical demands, compensation structures, and temporal arrangements in the occupation-health relationship.

Our analytical approach builds on recent methodological innovations in occupational epidemiology that emphasize the necessity of examining interconnected mediating pathways within contemporary work environments [65]. The findings extend current understanding of how occupational characteristics influence health outcomes in increasingly dynamic and digitalized labor markets.

### 4.5. Mediating mechanisms

Table 10 presents fixed-effects estimates with Driscoll-Kraay standard errors examining the mediating pathways between occupational characteristics and chronic health conditions. Our analysis employs advanced econometric techniques to address potential serial correlation and heteroskedasticity in panel data [66]. The baseline model reveals significant associations between occupational characteristics and chronic health conditions, with higher occupational status (β = -0.011, p < 0.05) and continuous employment (β = -0.005, p < 0.01) associated with lower chronic disease prevalence. Organizational type shows a positive association (β = 0.015, p < 0.01), aligning with recent evidence on occupational health gradients in contemporary labor markets [67].

**Table 10:** Analysis of the intrinsic mechanisms between occupation and chronic diseases.

	(1)	(2)	(3)	(4)	(5)
occupation	-0.011**	-0.011***	-0.011***	-0.013***	-0.011**
	(0.00)	(0.00)	(0.00)	(0.00)	(0.00)
cont_emp	-0.005***	-0.005***	-0.005***	-0.002***	-0.005***
	(0.00)	(0.00)	(0.00)	(0.00)	(0.00)
org_type	0.015***	0.015***	0.015***	0.018***	0.015***
	(0.00)	(0.00)	(0.00)	(0.00)	(0.00)
light	0.001				
	(0.00)				
moderate		0.004***			
		(0.00)			
heavy			0.000		
			(0.00)		
lnincome				0.003***	
				(0.00)	
labor_time					-0.004
					(0.00)
Control	Yes	Yes	Yes	Yes	Yes
Constant	0.774***	0.775***	0.775***	0.000	0.000
	(0.04)	(0.04)	(0.04)	(0.00)	(0.00)
Observations	4220	4220	4220	2815	4203
R-squared	0.453	0.453	0.453	0.393	0.448
Number of groups	4,126	4,126	4,126	2,777	4,111

***, **, and * indicate significance at the 1%, 5%, and 10% levels, respectively. Standard errors are reported in parentheses.

Our mediating analysis reveals several notable patterns. First, contrary to traditional assumptions about physical demands as primary mediators in occupation-health relationships [68], moderate work intensity shows significant mediating effect (β = 0.004, p < 0.01). Second, labor income demonstrates significant positive mediation (β = 0.003, p < 0.05), supporting recent theoretical work on income-health gradients in evolving labor markets [69]. This relationship potentially reflects the complex interplay between occupational status, compensation structures, and health outcomes in contemporary work environments.

Table 11 presents fixed effects estimates with Driscoll-Kraay standard errors examining how occupation affects self-rated health through three key mechanisms: physical labor, income, and working hours. The mediating effects reveal complex patterns. Physical labor shows no mediation, while income demonstrates a significant negative association (β = -0.030, p < 0.01), suggesting potential stress effects of high-earning positions. Working hours exhibit negative effect (β = -0.047, p < 0.10). These findings have important implications for workplace health interventions, highlighting the need for multifaceted approaches that address both direct occupational factors and their underlying mechanisms. The results contribute to our understanding of occupational health gradients and provide crucial insights for evidence-based policy design.

**Table 11:** Internal mechanisms between occupation and self-rated health.

	(1)	(2)	(3)	(4)	(5)
occupation	0.033***	0.034***	0.032***	0.020**	0.031***
	(0.00)	(0.00)	(0.00)	(0.00)	(0.00)
cont_emp	0.006	0.006	0.007	0.006	0.009
	(0.01)	(0.01)	(0.01)	(0.01)	(0.01)
org_type	-0.024**	-0.025*	-0.023*	-0.023	-0.028**
	(0.01)	(0.01)	(0.01)	(0.01)	(0.00)
light	-0.010				
	(0.02)				
moderate		0.016			
		(0.01)			
heavy			0.020		
			(0.01)		
lnincome				-0.029***	
				(0.00)	
labor_time					-0.047*
					(0.01)
Control	Yes	Yes	Yes	Yes	Yes
Constant	0.000	0.000	0.000	0.000	0.000
	(0.00)	(0.00)	(0.00)	(0.00)	(0.00)
Observations	10,805	10,805	10,805	5,346	10,626
R-squared	0.066	0.066	0.066	0.051	0.067
Number of groups	8,161	8,161	8,161	4,419	8,063

***, **, and * indicate significance at the 1%, 5%, and 10% levels, respectively. Standard errors are reported in parentheses.

The mediating mechanisms identified in this study explain approximately 60% of the total effects, which is comparable to or higher than similar studies in the field. For instance, their proposed mechanisms explained 45% of occupational effects on health outcomes [70], while a mediation proportion of 52% was reported in another study [71]. The remaining unexplained portion suggests several potential considerations. First, the theoretical framework and questionnaire design may limit our ability to capture all relevant mediating pathways. Some unmeasured mechanisms might include psychological factors (e.g., job satisfaction, workplace social support), environmental exposures (e.g., occupational pollutants), or lifestyle factors beyond those measured in the current study. Second, the complex nature of occupational health relationships may involve interaction effects or non-linear relationships that our current analytical framework does not fully capture. Future research could benefit from incorporating more comprehensive mediating variables and employing advanced statistical methods to examine potential interaction effects and non-linear relationships.

Table 12 presents the interaction terms of BMI index. The regression results of the core independent variables remain unchanged. It is worth noting that BMI increases the probability of having chronic disease but lower the self-rated health. Using chronic diseases as the dependent variable. The interaction of occupation type and BMI shows significant positive effect (β = 0.001, p < 0.05). BMI index weakens the promoting effect of higher occupational types on chronic diseases. The interaction of continuous employment and BMI shows significant negative effect (β = -0.002, p < 0.05). BMI index enhance the promoting effect of higher continuous employment on chronic diseases. The interaction of organization type and BMI shows significant negative effect (β = -0.002, p < 0.01). BMI index weakens the reducing effect of public sector work on chronic diseases.

**Table 12:** Interaction terms of BMI index.

	chronic			health_self		
	(1)	(2)	(3)	(4)	(5)	(6)
occupation	-0.012**	-0.010**	-0.010**	0.034***	0.034***	0.034***
	(0.00)	(0.00)	(0.00)	(0.00)	(0.00)	(0.00)
cont_emp	-0.005***	-0.003***	-0.005***	0.006	0.006	0.006
	(0.00)	(0.00)	(0.00)	(0.01)	(0.01)	(0.01)
org_type	0.009***	0.010***	0.012***	-0.023*	-0.023*	-0.026*
	(0.00)	(0.00)	(0.00)	(0.01)	(0.01)	(0.01)
bmi	0.011***	0.011***	0.011***	-0.005*	-0.005	-0.005*
	(0.00)	(0.00)	(0.00)	(0.00)	(0.00)	(0.00)
occupation*bmi	0.001**			-0.001*		
	(0.00)			(0.00)		
cont_emp*bmi		-0.002**			0.001	
		(0.00)			(0.00)	
org_type*bmi			-0.002***			0.008***
			(0.00)			(0.00)
Control	Yes	Yes	Yes	Yes	Yes	
Constant	0.612***	0.595***	0.610***	0.000	1.200***	0.000
	(0.02)	(0.02)	(0.03)	(0.00)	(0.04)	(0.00)
Observations	4,220	4,220	4,220	10,805	10,805	10,805
R-squared	0.464	0.464	0.464	0.067	0.067	0.067
Number of groups	4,126	4,126	4,126	8,161	8,161	8,161

***, **, and * indicate significance at the 1%, 5%, and 10% levels, respectively. Standard errors are reported in parentheses.

Using self-rated health as the dependent variable. The interaction of occupation type and BMI shows significant negative effect (β = -0.001, p < 0.1). BMI index weakens the reducing effect of higher occupational types on self-rated health. The interaction of continuous employment and BMI shows no significant effect. The interaction of organization type and BMI shows significant positive effect (β = 0.008, p < 0.01). BMI index weakens the promoting effect of public sector work on self-rated health.

### 4.6. Robustness checks

To validate the robustness of the main findings, two methods for robustness testing were employed, the first using alternative samples and the second using alternative variables. Alternative samples utilize the China Family Panel Studies (CFPS) dataset covering 2014, 2016, and 2018. Table 13 presents fixed-effects estimates with Driscoll-Kraay standard errors for both CHNS and CFPS samples. The CFPS results largely corroborate our baseline findings from CHNS. First, occupational status maintains significant associations with health outcomes, showing negative effects on chronic disease incidence (β = -0.002, p < 0.05) and self-reported health (β = -0.004, p < 0.05). Second, employment type demonstrates a negative association with chronic disease (β = -0.011, p < 0.10) and a positive relationship with self-reported health (β = 0.064, p < 0.05), confirming that higher-level employment categories are associated with lower health outcomes.

**Table 13:** Robust test by using alternative samples.

VARIABLES	Chronic	health_self
occupation	-0.002**	-0.004**
	(0.00)	(0.00)
cont_emp	-0.011*	0.064**
	(0.00)	(0.01)
org_type	0.011*	-0.037**
	(0.00)	(0.01)
age	0.000	-0.047***
	(0.00)	(0.00)
age2	0.000**	0.000***
	(0.00)	(0.00)
gender	-0.016***	0.183***
	(0.00)	(0.01)
marriage	-0.004	0.056***
	(0.00)	(0.00)
edu	-0.001	0.015**
	(0.00)	(0.00)
smoke	-0.024***	0.034**
	(0.00)	(0.00)
drink	-0.037***	0.137***
	(0.00)	(0.00)
sleep	-0.003*	0.032***
	(0.00)	(0.00)
urban	0.009**	-0.047*
	(0.00)	(0.01)
AREA1	0.016***	-0.063**
	(0.00)	(0.01)
AREA2	0.000	0.026***
	(0.00)	(0.00)
T1	-0.005**	0.033***
	(0.00)	(0.00)
T2	0.035	4.234***
	(0.02)	(0.04)
Constant	0.036	4.141***
	(1.89)	(88.85)
Observations	52,300	52,300
R-squared	0.051	0.090
Number of groups	26,928	26,928

***, **, and * indicate significance at the 1%, 5%, and 10% levels, respectively. Standard errors are reported in parentheses.

Organizational type effects also remain consistent across datasets, showing negative associations with chronic disease (β = -0.011, p < 0.10) and negative relationships with self-reported health (β = -0.037, p < 0.05). These findings align with our baseline CHNS results, where organizational type similarly affects health outcomes.

The consistency of these relationships across different datasets strengthens the reliability of our findings and suggests that the identified associations between occupational characteristics and health outcomes are robust to alternative sampling frames and time periods.

Table 14 presents the robust test using alternative variables. Two alternative variables were employed. The first one is two-category self-rated health, where a value of 1 indicates “Good” self-rated health, and 0 indicates otherwise. The second involves calculating the relative deprivation index of self-rated health. The results of the core independent variables remain unchanged. The result demonstrates robustness.

**Table 14:** Robust test by using alternative variables.

VARIABLES	Health_self (Good/Not Good)	RD
occupation	-0.027***	-0.007***
	(0.00)	(0.00)
cont_emp	-0.007	-0.002
	(0.00)	(0.00)
org_type	0.028**	0.007**
	(0.00)	(0.00)
age	-0.005*	-0.001*
	(0.00)	(0.00)
age2	-0.000	-0.000
	(0.00)	(0.00)
gender	0.039	0.009
	(0.02)	(0.00)
marriage	0.032	0.008*
	(0.01)	(0.00)
edu	0.016*	0.005*
	(0.00)	(0.00)
smoke	-0.007	-0.002
	(0.01)	(0.00)
drink	0.020**	0.005***
	(0.00)	(0.00)
sleep	0.000	0.000
	(0.00)	(0.00)
urban	-0.053	-0.013
	(0.02)	(0.01)
AREA1	0.134*	0.035*
	(0.03)	(0.01)
AREA2	0.078*	0.020*
	(0.02)	(0.01)
T1	0.901***	0.251***
	(0.01)	(0.00)
T2	0.917***	0.251***
	(0.01)	(0.00)
Constant	0.836***	0.250***
	(0.02)	(0.01)
Observations	10,805	10,805
R-squared	0.066	0.063
Number of groups	8,161	8,161

***, **, and * indicate significance at the 1%, 5%, and 10% levels, respectively. Standard errors are reported in parentheses.

### 4.7. Endogeneity tests

This study identifies three main types of endogeneity concerns in the relationship between occupational characteristics and health outcomes. First, there exists reverse causality, where individual health status may influence occupational choices and mobility. As shown in Table 4, while occupational status significantly affects chronic disease incidence (β = -0.011, p < 0.05) and self-rated health (β = 0.035, p < 0.01), these correlations may partly stem from healthier individuals being more likely to attain and maintain higher occupational positions. Second, omitted variable bias may arise from unobservable individual characteristics (e.g., ability, personality traits) that simultaneously influence both occupational choices and health outcomes. This is evidenced by the significant effects of control variables in Table 4, such as education’s impact on both chronic diseases (β = -0.017, p < 0.01) and self-rated health (β = -0.020, p < 0.1).

To address these endogeneity concerns, we employ housing prices as an instrumental variable. The choice is justified on several grounds: First, evidence shows that a 10% increase in housing prices corresponds to a 1.2 percentage point rise in high-skill occupation employment share [72]. Other research finds that housing prices significantly affect labor participation decisions through household wealth effects, with a 1% housing price increase raising labor force participation by 0.3% [73]. Second, regarding the exogeneity condition, studies demonstrate that housing price variations are primarily driven by macro factors such as land supply, monetary policy, and population mobility [74]. Recent evidence further confirms that individual health status has negligible impact on regional housing prices, supporting the exogeneity assumption of housing prices as an instrument [75]. Third, concerning the exclusion restriction, research shows that housing prices primarily affect individual outcomes through occupational choices and income channels rather than direct health pathways [76].

In the Chinese context, housing prices demonstrate particular validity as an instrumental variable. Based on China Household Finance Survey data, research indicates that housing prices not only influence individual occupational choices but also affect occupational mobility through household wealth and intergenerational resource transfers [77]. Evidence from urban China finds that housing price fluctuations significantly impact employment structure and occupational choices, particularly in first- and second-tier cities, where high housing prices drive workers toward higher-income occupations [78].

Table 15 presents the two-stage least squares (2SLS) estimation results. After controlling for endogeneity, occupational status shows stronger effects on both chronic disease incidence (β = -0.608, p < 0.01) and self-rated health (β = 1.170, p < 0.05). Employment contract type similarly demonstrates significant effects on chronic diseases (β = 1.713, p < 0.05) and self-rated health (β = 0.510, p < 0.01), while organization type shows contrasting effects between chronic diseases (β = 2.105, p < 0.01) and self-rated health (β = -1.237, p < 0.01). The models maintain robust control for demographic and socioeconomic factors, with education showing consistent effects (β ≈ -0.64 for chronic diseases, p < 0.01) and urban residence demonstrating persistent influence (β ≈ -0.17 to -0.66, p < 0.01). Age effects exhibit nonlinear patterns, particularly in chronic disease models.

**Table 15:** Effects of occupation on health-2SLS regression results.

		Chronic		Health_self		
VARIABLES	(1)	(2)	(3)	(4)	(5)	(6)
occupation	-0.6080***			1.1696**		
	(-4.45)			(2.06)		
cont_emp		1.7125**			0.5096***	
		(2.08)			(3.80)	
org_type			2.1050***			-1.2369***
			(4.07)			(-4.02)
age	-0.0357***	-0.0116	-0.0596***	0.0688**	0.0109	0.0346***
	(-2.97)	(-0.48)	(-3.13)	(2.09)	(1.34)	(3.25)
age2	0.0005***	0.0004	0.0006***	-0.0007*	0.0000	-0.0003**
	(3.60)	(1.36)	(3.30)	(-1.89)	(0.14)	(-2.34)
gender	-0.0107	0.0845	0.0090	0.1281	-0.0306	-0.0011
	(-0.31)	(1.00)	(0.21)	(1.45)	(-0.98)	(-0.04)
marriage	0.0004	-0.0536	-0.0249	0.0319	-0.0013	0.0350
	(0.01)	(-0.58)	(-0.55)	(0.60)	(-0.04)	(1.16)
edu	-0.6378***	-0.1773*	-0.6476***	1.1974**	-0.1323***	0.3190***
	(-4.44)	(-1.88)	(-4.04)	(1.96)	(-5.38)	(3.33)
smoke	0.0408	-0.0368	0.0104	-0.1069	-0.0077	-0.0133
	(1.12)	(-0.43)	(0.24)	(-1.42)	(-0.24)	(-0.44)
drink	-0.0629*	0.0043	-0.0011	0.0761	-0.0308	-0.0150
	(-1.72)	(0.05)	(-0.03)	(1.01)	(-1.03)	(-0.52)
sleep	0.0155	0.0279	-0.0002	-0.0475**	-0.0157	-0.0196*
	(1.13)	(0.77)	(-0.01)	(-1.98)	(-1.19)	(-1.68)
urban	-0.1744***	-0.6645**	-0.1944***	0.4299**	-0.1836***	0.1669***
	(-3.74)	(-2.06)	(-3.25)	(2.13)	(-3.10)	(3.78)
AREA1	-0.0777	-0.1693	-0.0827	0.1118	-0.2991***	-0.1178***
	(-1.63)	(-1.11)	(-1.46)	(0.69)	(-7.73)	(-3.24)
AREA2	-0.0028	0.0366	-0.0289	-0.0137	-0.1183***	-0.0673**
	(-0.09)	(0.41)	(-0.70)	(-0.19)	(-3.63)	(-2.08)
_cons	3.7421***	-3.3586**	1.9255***	-5.9929	0.4935	0.4562
	(4.14)	(-2.07)	(3.25)	(-1.64)	(1.44)	(1.36)
N	3381	3381	3381	4301	4301	4301

***, **, and * indicate significance at the 1%, 5%, and 10% levels, respectively. Standard errors are reported in parentheses.

These 2SLS estimates reveal larger effect magnitudes compared to ordinary least squares approaches, suggesting that failing to account for endogeneity may understate the true impact of occupational characteristics on health outcomes. The consistency across multiple health measures strengthens the causal interpretation of these relationships, providing more reliable evidence for understanding the occupation-health nexus.

## 5. Discussion

Drawing on the empirical analysis of CHNS panel data from 2004 to 2015, this study reveals several important findings that warrant detailed discussion.

First, the significant correlation between occupational characteristics and health deepens our understanding of the fundamental relationship between occupational stratification and health inequality [1–3]. The study finds that better employment relationships and stable occupational types actually increase the risk of chronic diseases, creating an interesting contrast with previous research on occupational status and health risks [8–10]. This seemingly contradictory phenomenon can be explained through occupational exposure theory [11–12]: better occupations often involve long-term desk work and high-intensity mental stress, factors closely related to cardiovascular disease risks [15,16]. Meanwhile, based on the effort-reward imbalance theory [17], the rewards of better occupations, such as high income and social status, may offset the negative impact of health risks on self-rated health, explaining the discrepancy between objective and subjective health evaluations.

Second, gender heterogeneity analysis reveals the importance of institutional environment. Males show stronger positive effects in occupational health [20,21], a phenomenon particularly evident in transitional economies. Health disparities under different ownership structures [29,30] interweave with gender role expectations, forming unique gender stratification patterns. This indicates that gender differences in occupational health stem not only from physiological characteristics but also reflect deeper institutional arrangements shaping gender roles.

Third, public sector employment shows a trend contrary to general expectations: lower labor intensity is associated with higher chronic disease risk. This finding supplements existing research on ownership structure and health inequality [31–33], providing a new explanatory framework for understanding how institutional characteristics influence health through work arrangements. This paradoxical relationship suggests we need to rethink the complex interactions between work intensity, institutional protection, and health risks.

Fourth, this study reveals a significant negative correlation between urban residence and chronic diseases, reflecting a unique phenomenon in China’s urbanization process. This seemingly paradoxical relationship can be understood through three mechanisms: First, urban areas provide better access to medical resources and stronger health awareness. As Chen et al. demonstrated, China’s new-type urbanization has made significant progress in public services such as healthcare, with urban residents showing marked improvements in access to preventive healthcare services [54]. Second, the “healthy migrant effect” leads to population self-selection. Mou et al.’s systematic review supports this view, noting that health selection effects play a crucial role in China’s rural-urban population mobility [5]. Third, urban areas have more comprehensive occupational health protection systems. Liu et al. confirmed that more developed healthcare systems in urban areas positively impact residents’ health [56].

These findings not only complement existing research on urban-rural health disparities but also provide a new explanatory framework for understanding how institutional environments influence health outcomes through urban-rural segmentation. This suggests the need for future research to further examine the impact of urban-rural disparities in healthcare resource allocation, regional differences in occupational health policy implementation, and patterns of health behaviors across urban and rural settings.

## 6. Research contributions and limitations

In terms of theoretical contributions, this study enriches the theoretical framework of occupational health research by discovering the “dual effect” mechanism, transcending the traditional unidirectional assumption between occupation and health. Methodologically, it innovatively adopts a multi-level analytical framework, combining micro-individual data with macro-institutional data, and effectively addresses endogeneity through instrumental variables. Empirically, it systematically examines health differences across employment types, employment relationships, and organizational types, providing rich empirical evidence for understanding occupational stratification and health inequality.

The study has several main limitations: First, although it uses CHNS panel data from 2004–2015 and conducts robustness checks through CFPS data, the measurement indicators for occupational psychological factors (such as work stress and occupational identity) are relatively lacking. Second, while the mediation effect analysis explains about 60% of the total effect, about 40% of the effect mechanisms remain uncaptured by the existing framework. Third, the analysis of differentiated policy needs for different groups could be further deepened, and the cost-benefit analysis of policy implementation needs strengthening.

## 7. Conclusions

Based on empirical analysis, this study reaches three core conclusions: Occupational health shows significant “dual effects,” where better occupational status simultaneously improves self-rated health while increasing chronic disease risks, with work intensity playing an important mediating role; institutional environment significantly influences health outcomes through work arrangements, with public sector institutional characteristics (such as stable job security and standardized working hours) moderating the relationship between occupation and health; occupational health effects show notable gender differences, with women facing greater health risks at the same occupational status, mainly due to institutional factors such as work-family conflicts.

Based on these conclusions, this study proposes the following detailed policy recommendations:

First, implement gender-differentiated occupational health policies. This includes establishing comprehensive health risk screening programs tailored to gender-specific needs, such as regular physical examinations, mental health assessments, and reproductive health monitoring. A flexible work system should be implemented with clear guidelines, covering remote work options and adjustable working hours to help employees balance work and family responsibilities. Support measures should include standardized workplace childcare facilities with specific operational guidelines and quality standards, as well as professional counseling services.

Second, strengthen public sector health management reform. Health indicators should be integrated into performance evaluation systems, accounting for 20–30% of overall assessment, including metrics for both physical and mental well-being. A mandatory rest program should be implemented through automated work schedule monitoring systems to prevent overwork. Structured stress management projects should be developed, comprising regular workshops, professional counseling services, and organized stress reduction activities.

Finally, enhance the multi-level occupational health protection system. This requires strengthening legislative oversight through regular workplace inspections and clear enforcement mechanisms. Detailed workplace health and safety standards should be established with specific implementation timelines and assessment criteria. Social security reforms should focus on providing comprehensive health coverage and occupational disease protection, with special attention to gender-specific health needs and preventive care.
